# Gambling disorder in late life: A systematic review of behavioral patterns, clinical correlates, and psychotherapeutic interventions

**DOI:** 10.1111/ajad.70188

**Published:** 2026-07-18

**Authors:** Graziella C. Prezzavento, Maria C. Quattropani, Vittorio Lenzo, Alberto Sardella, Chiara Franceschini, Giulia Galea, Pasquale Caponnetto

**Affiliations:** ^1^ Department of Educational Sciences Section of Psychology, University of Catania Catania Italy; ^2^ Department of Clinical and Experimental Medicine Center of Excellence For The Acceleration of Harm Reduction (COEHAR), University of Catania Catania Italy

## Abstract

**Background and Objectives:**

Gambling in older adults represents an emerging but still underrecognized mental health concern. Although gambling participation is increasingly observed in later life, the specific psychological, social, cognitive, and biological factors associated with gambling‐related problems in older adults remain insufficiently characterized.

**Methods:**

A systematic review was conducted in accordance with PRISMA guidelines. Empirical studies involving adults aged ≥55 years with Gambling were identified through searches of Web of Science, PubMed/MEDLINE, Scopus, PsycNET, ClinicalTrials.gov, and WHO ICTRP from January 15 to June 15, 2025.

**Results:**

From 40,263 identified records, 35 studies met the inclusion criteria. The included studies, published between 2000 and 2025, primarily investigated gambling behaviors, severity, psychosocial correlates, and associated mental health outcomes in older adults. Commonly identified correlates to gambling‐related problems included loneliness, reduced social support, financial difficulties, male gender, stressful life events, and early gambling initiation. Gambling was frequently reported as a recreational activity but could become maladaptive when used as a strategy to regulate negative emotions or cope with social isolation. Psychiatric comorbidity, particularly depression and anxiety, was frequently observed in clinical and high‐risk samples. Evidence regarding psychological interventions remains limited but suggests potential benefits.

**Discussion and Conclusions:**

Current evidence highlights substantial gaps in age‐specific assessment and targeted interventions. Future studies should prioritize tailored psychological interventions addressing loneliness, emotional regulation, cognitive distortions, and life transitions in later adulthood.

## BACKGROUND AND OBJECTIVES

Gambling Disorder remains the only formally recognized non‐substance‐related addictive disorder, defined by the DSM‐5 as a pattern of persistent and recurrent problematic gambling behavior causing significant impairment or distress.^1^ Diagnosis requires meeting at least four out of nine criteria within a 12‐month period, including preoccupation with gambling, failed attempts to cut back, use of gambling to cope with negative emotions, chasing losses, and continued gambling despite serious adverse consequences. Importantly, DSM‐5 diagnostic criteria are agnostic with respect to the precipitating factors or motivations underlying gambling behavior: if the criteria are met, the disorder is present regardless of whether gambling was initiated for social, emotional, or recreational reasons. This distinction is clinically relevant when assessing older adults, whose motivational pathways to gambling may differ from younger populations without this implying a lesser degree of psychopathological severity. In recent years, gambling participation is increasing significantly among the older people and becoming a particularly widespread and regular recreational behavior among this population.^2^

Estimating the true prevalence of gambling disorder in later life, however, remains methodologically challenging. The wide range reported in the literature, from 0.01% to 10.6% for adults aged 60 and above, reflects substantial heterogeneity in measurement instruments, age thresholds, cultural contexts, and sampling strategies, and should therefore be interpreted with considerable caution rather than as a reliable epidemiological estimate.^3^ Factors contributing to this vulnerability include social isolation, cognitive decline, and financial insecurity. These factors, however, are not exclusive to older adults; younger problem gamblers may present similar vulnerabilities. What distinguishes the older adult context is the convergence and co‐occurrence of multiple age‐specific transitions (retirement, bereavement, functional decline, reduced social networks, and increased unstructured leisure time) which together may create a particularly fertile environment for gambling escalation in this population, even when individual risk factors are shared with younger age groups.

A further biological vulnerability specific to older adults deserves explicit consideration. Neurobiological changes associated with aging, including dopaminergic system decline, reduced frontal inhibitory control, and altered reward processing, may increase susceptibility to reinforcement‐based behaviors such as gambling.^4^ Moreover, dopamine agonist medications, including pramipexole and ropinirole, prescribed for Parkinson's disease and restless leg syndrome, have been well documented as pharmacological precipitants of impulse control disorders including gambling disorder in older adults.^5^ Clinicians assessing gambling in older adults should routinely screen for current dopaminergic medication use as a potential etiological factor.

Several theoretical frameworks have been proposed to explain the emergence and maintenance of this behavioral addiction across the lifespan, and their application to older populations deserves explicit consideration. The Pathways Model of Problem Gambling proposed by Blaszczynski and Nower conceptualizes gambling disorder as the outcome of distinct vulnerability trajectories, including behaviorally conditioned, emotionally vulnerable, and impulsive–antisocial pathways. This model emphasizes heterogeneity in etiological mechanisms and may be particularly relevant for older adults, whose psychological profiles and life experiences differ substantially from younger populations.^6^ From a lifespan perspective, Socioemotional Selectivity Theory posits that as individuals perceive their remaining time as limited, motivational priorities shift toward emotionally meaningful goals and regulation of affective states.^7^ While boredom, social isolation, and emotional dysregulation may drive gambling engagement across all age groups, in older adults these motivations are amplified by the structural conditions of later life, including retirement‐related loss of routine and identity, bereavement, and reduced access to alternative social activities. Emotional vulnerability arising from these losses may drive older adults toward gambling as a strategy to escape negative emotions such as loneliness or depression, and to restore a sense of well‐being.^8^ Tira et al. identify three pathways to gambling disorder: the grief pathway, driven by negative life events and emotional distress; the habit pathway, in which repeated exposure to gambling fosters erroneous beliefs (gambler's fallacy, near‐miss effects) and escalating behavior, with boredom shifting from a reason to start gambling to an excuse to continue; and the dormant pathway, rooted in predispositions to compulsive behavior, low self‐control, and strong comorbidities such as tobacco and alcohol dependence.^9^ Stress–vulnerability models suggest that addictive behaviors may emerge from the interaction between predisposing individual characteristics and environmental stressors.^10^ In older adults, life transitions such as retirement, bereavement, declining health, or financial insecurity may act as stressors that interact with individual vulnerabilities (e.g., impulsivity, emotional dysregulation), increasing the risk for maladaptive behavioral engagement.

Despite the growing prevalence of gambling in older adults and its significant impact on quality of life, psychological well‐being, and healthcare utilization, research in this population remains limited.

This systematic review aims to address this gap by pursuing three explicit objectives: (1) to characterize the prevalence and severity of gambling disorder and problematic gambling behaviors in adults aged 55 years and older; (2) to identify the psychological, cognitive, social, and biological correlates associated with gambling in this population, with particular attention to risk and protective factors; and (3) to evaluate the available evidence on psychological and psychotherapeutic interventions targeting this condition in later life. We hypothesize that gambling‐related problems would be associated with a combination of psychosocial vulnerabilities (e.g., loneliness, reduced social support, stressful life events), psychiatric symptoms, and biological contributors rather than with aging alone, and that the functional role of gambling as a coping strategy will emerge as a central mediating mechanism. These hypotheses guided both the extraction and the interpretation of the evidence.

## METHODS

This systematic review was recorded on PROSPERO (CRD42025639009) and conducted according to the Preferred Reporting Items for Systematic Reviews and Meta‐Analyses (PRISMA 2020). The review focused on the following research question: “How do psychosocial vulnerabilities and coping‐related mechanisms contribute to Gambling Disorder in adults aged 55 years and older, and which evidence‐based psychological or psychotherapeutic interventions effectively address these mechanisms in prevention and treatment?”.

### Eligibility criteria

Studies were selected according to the PICOs framework (Population, Intervention, Comparator, Outcomes), with an additional criterion for Study Design:

#### Population (P)

- Inclusion: adults aged ≥55 years diagnosed with or presenting symptoms of Gambling Disorder or Problem Gambling (DSM‐5, ICD‐11, SOGS, PGSI, or equivalent); studies comparing older and younger adults are eligible if gambling outcomes are reported separately for the older cohort.
- Exclusion: participants aged <55 years with no older adult subgroup; studies focused on substance use disorders or other behavioral addictions unrelated to gambling.

#### Intervention (I)

- Inclusion: psychological or psychotherapeutic interventions (e.g., CBT, Motivational Interviewing, brief interventions, acceptance‐based therapies, psychoeducational programs).
- Exclusion: pharmacological‐only treatments with no psychological component.

#### Comparison (C)

- Inclusion: waitlist control, TAU, active control, or alternative psychological intervention; pre‐post single‐group designs are also eligible given the scarcity of controlled trials in this population.
- Exclusion: purely correlational designs reporting only statistical associations with no clinical reference.

#### Outcome (O)

- Inclusion: studies reporting measurable gambling‐related outcomes (severity, frequency, money wagered, urges, cognitive distortions); secondary outcomes (depression, anxiety, quality of life) also considered.
- Exclusion: studies without quantifiable gambling‐related outcomes following intervention.

#### Study design (S)

- Inclusion: peer‐reviewed empirical studies including RCTs, quasi‐experimental designs, cross‐sectional studies, single‐case experimental designs, and clinical case studies.
- Exclusion: nonempirical publications (commentaries, editorials, narrative reviews, opinion pieces); studies with insufficient methodological detail or without full‐text access; articles published in languages other than English.

Although no consensus exists on the age threshold for “older adulthood,” with gambling studies ranging from 50 to 70 years, we adopted a cutoff of 55 years, consistent with prior gambling addiction literature and empirical evidence identifying this age as a meaningful transition point (onset of regular gambling in older women; beginning of age‐related decision‐making impairment). This threshold was established a priori to ensure adequate coverage of the available evidence. We acknowledge that the 55+ category encompasses heterogeneous subgroups (“young‐old” 55–69; “older‐old” 70+) that may differ in cognitive and functional status, which may affect the interpretability of findings.

### Information sources

The following information sources were used to identify studies for inclusion in this review:

#### Databases

Web of Science (WOS), PubMed/MEDLINE, Scopus, PsycNET.

#### Registers

ClinicalTrials.gov, WHO International Clinical Trials Registry Platform (ICTRP).

#### Reference lists

Screening of reference lists from included studies and relevant reviews.

### Search strategy

A comprehensive search strategy was developed for each database and source (Supporting Information S1: Table 1) and conducted between January 15 and June 15, 2025. No separate update search was performed.

### Selection process

The selection process followed these steps:

#### Screening of titles and abstracts

Three independent reviewers (G.C.P, G.G., C.F.) screened all records identified through database searches to exclude irrelevant studies.

#### Full‐text review

Full‐text articles of potentially eligible studies were retrieved and independently assessed by the same reviewers.

#### Consensus and resolution

Disagreements between reviewers were resolved through discussion or, if necessary, consultation with supervisors (M.C.Q., V.L., A.S., P.C.).

#### Automation tools

Rayyan was used for screening and deduplication.

### Data collection process

Data were collected from eligible reports using a standardized data extraction form (Table 1). Three reviewers (G.C.P., C.F., G.G.) independently extracted data to ensure accuracy and completeness. Any discrepancies were resolved through discussion or consultation with supervisors (M.C.Q., V.L., A.S., P.C.).

**Table 1:** Characteristics of the included studies.

Authors	Nation	Study design	Sample	Intervention/treatment	Measurements	Outcome
McNeilly D. P. & Burke W. J., 2000^11^	USA	Cross‐sectional	315	NA	SOGS‐R; GDS‐15; SWLS; background demographic questions, and items generated by the author to assess attitudes and motivations for gambling	Two groups were compared: active patrons of bingo parlors and casino day trips, and community‐dwelling older adults not actively involved in gambling. Both groups were predominantly retired Caucasian females with at least a high school education and income $15,000–$34,999, though they differed in age (community group x̄ = 78.2 years), marital status, and residence. Comparisons were made on depression, life satisfaction, gambling behaviors, motivations, attitudes, and demographics, with no significant differences emerging on depression (GDS) or life satisfaction (SWLS). Gambling patrons showed the highest frequency of gambling across all types and higher per‐session expenditure (lottery $2.27; bingo $12.16; casino $53.12). They also showed significantly higher SOGS‐R scores (*p* < .001), with 11% scoring ≥5 (probable pathological gambling) vs. 2.7% in the community group, and greater prevalence of problematic behaviors including gambling more than intended (46.2%), guilt (20.9%), borrowing from credit cards (11.3%), and arguing over money (19.8%). Primary motivations among patrons were relaxation/fun (68.1%), getting away for the day (43.7%), passing time (42.7%), inexpensive meals (42%), and relieving boredom (30%), providing an initial profile of older adults' attitudes, motivations, and gambling behaviors.
Bazargan M. et al., 2001^12^	USA	Cross‐sectional	80	NA	SOGS; BSI; 16 items measure of negative stressful life‐events	The sample had a mean age of 69 years (range 60–91), was predominantly female (80%), mostly unmarried (widowed 36%, divorced/separated/never married 33%, married 31%), with low educational attainment (30% below ninth grade, 35% high school) and poor‐to‐fair health (36% fair/poor, 52% good; 52% hypertension, 16% diabetes). Gambling severity (SOGS range 0–17, mean 2.6, SD 3.3) classified 64% as non/occasional gamblers, 19% as light‐to‐moderate, and 17% as heavy‐to‐pathological. Heavy/pathological gamblers showed significantly lower psychological well‐being (*F* = 4.92, *p* < .01), higher anxiety (*F* = 3.9, *p* < .05), more obsessive‐compulsive symptoms (*F* = 7.6, *p* < .001), and a greater number of stressful life events (4.3 vs. 2.4; *F* = 3.7, *p* < .05), particularly changes in residence, financial difficulties, marital separation, family conflicts, and arguments with friends/neighbors. No association with depression was found. Heavy/pathological gamblers also reported lower perceived health status, lower perceived control over future health, and lower religiousness. No association with depression was detected.
Ladd G. T. et al., 2003^13^	USA	Cross‐sectional	492	NA	SOGS	91.7% of participants reported lifetime gambling experience 100% from bingo events and 88.6% from senior centers. Of all participants (*n* = 492), 89.4% were non‐problem gamblers (SOGS 0‐2), 5.9% were problem gamblers (SOGS 3‐4), and 4.7% were pathological gamblers (SOGS 5‐14). Problem/pathological gamblers were younger (*p* < .01), more likely male (*p* < .001), and showed greater gambling frequency and expenditures in both past‐year and past‐two‐month timeframes (all *p* < .001) compared to non‐problem gamblers.
Burge A. N., 2004^14^	USA	Cross‐sectional	52	NA	SOGS; ASI; SF‐36	The sample had a mean age of 67 ± 7 years, 14 ± 3 years of education, mean income $35,054 ± 23,121, was predominantly white (96%), with 44% married and 21% widowed. Participants were classified by gambling onset: early versus late. Early‐onset gamblers showed significantly higher ASI gambling, medical, and psychiatric scores (*F* = 2.94, *p* < .05), gambled more frequently, and preferred card games (54% vs. 21%, *p* < .05) and sports betting (43% vs. 0%, *p* < .05). They were also more likely to have chronic medical conditions and to be receiving psychiatric treatment. Physical health (SF‐36) was significantly lower in early‐onset gamblers (46.4 ± 2.2 vs. 53.2 ± 2.3, *p* < .05), with worse scores across physical functioning, role‐physical, bodily pain, social functioning, and mental health subscales; mental health summary scores did not differ significantly between groups.
‍Kaush O., 2004^15^	USA	Retrospective observational study	135	NA	SCID‐IV; ASI; The Barratt Impulsivity Scale GSRI	The elderly subsample (*N* = 37, 97% male, mean age 65.8 ± 5.4 years) was compared to a younger cohort (mean age 46.7 ± 8.1 years). Beyond age and employment status (elderly more likely retired, *χ* ^2 ^= 31.1, *p* < .0001; less likely on disability), demographic composition did not differ significantly between groups. Duration of problem gambling and choice of gambling activities were comparable, with slot machines being the most common problematic form in both cohorts. Over 25% of elderly gamblers had developed a gambling problem within 5 years of admission, and nearly 50% within 10 years. Elderly gamblers reported significantly less lifetime emotional and physical abuse, lower rates of lifetime alcohol and drug abuse/dependence, and fewer suicide attempts, though suicidal ideation history was comparable between groups. ASI composite scores were largely similar across cohorts, with the exception of psychiatric severity scores, which were significantly higher in younger gamblers despite no significant difference in clinical psychiatric diagnoses. Impulsivity (Barratt scores) did not differ between groups. Employment severity scores were equivalent despite differing employment status, likely reflecting gambling debts exceeding retirement income among elderly gamblers.
‍Desai R. A. et al., 2004^16^	USA	Cross‐sectional	2417	NA	NIMH DIS	Among older adults (*N* = 388), 50.3% reported past‐year gambling versus 65.5% of younger adults, with significantly lower rates of problem/pathological gambling (0.50% vs. 2.43%, *p* < .02). Excluding problem gamblers, older recreational gamblers were more likely than older nongamblers to be male, more educated, and employed full‐time. After sociodemographic adjustment, older recreational gamblers were significantly more likely to report good/excellent subjective health (OR = 2.49, 95% CI = 1.46–4.25, *p* = .0008), past‐year alcohol use (*p* < .05), and marginally more likely to have a lifetime history of depression (*p* = .10). Compared to younger recreational gamblers, older gamblers were less likely to have initiated gambling before age 18 (*p* < .002) and less likely to gamble only a few days per year (*p* < .03), but more likely to gamble one to several times per week (*p* < .005) or daily (*p* < .008), and more likely to have won >$500 (*p* < .02). Good‐to‐excellent subjective health was more strongly associated with recreational gambling in older than younger adults (*p* < .02).
Vander Bilt J. V. et al., 2004^17^	USA	Cross‐sectional and Longitudinal study	1681	NA	Self‐report measure for gambling; CES‐D; MMSE; Self‐rated health, social support, alcohol use, smoking.	Participants were followed longitudinally across three waves (1993–2001), with complete data available for a subset at wave 6. Among those leaving home, gambling/bingo was the least frequent outside activity (47.7%). Cross‐sectionally, gambling was significantly associated with younger age (gamblers averaged 2 years younger than nongamblers: 77.5 vs. 79.5, *p* < .0001), male gender, fewer years of education, greater social support, lower depression scores, better self‐rated health, past‐year alcohol use, and higher cognitive functioning. In multivariate logistic regression, younger age (70–79), greater social support (OR = 2.7, 95% CI = 1.59–4.57), and past‐year alcohol use remained independently associated with gambling. Longitudinally, the same predictors at wave 4, younger age, male gender, greater social support, alcohol use, and prior gambling activity, significantly predicted gambling at wave 6. Attrition analyses confirmed no significant association between wave 4 gambling and dropout or mortality, ruling out selection bias.
‍Erickson L. et al., 2005^18^	USA	Cross‐sectional	343	NA	SOGS; SF‐12	Among the sample, 89.8% were non‐problem gamblers (SOGS 0–2, M = 0.4, SD = 0.7), 6.4% problem gamblers (SOGS 3–4, M = 3.3, SD = 0.5), and 3.8% pathological gamblers (SOGS ≥ 5, M = 7.7, SD = 2.1), with no significant demographic differences across groups. About two‐thirds of pathological gamblers and all problem gamblers reported recent gambling activity, with higher recent expenditures than non‐problem gamblers. In multivariate analyses, age was inversely associated with physical health (*p* < .001), while gender was not significant. After controlling for these variables, gambling severity was a significant predictor of SF‐12v2 scores (*F* = 2.51, *p* < .001), with problem/pathological gambling associated with significantly poorer general health (*p* < .05), social functioning (*p* < .05), and role‐emotional functioning (*p* < .05). No significant differences emerged for physical functioning, role‐physical, vitality, or mental health subscales.
Wiebe J. M., & CoxB. J., 2005^19^	Canada	Cross‐sectional	1000	NA	Telephone interviews; SOGS‐R	Of the total sample, 77.5% had gambled in the past year, with 1.6% classified as problem gamblers and 1.2% as probable pathological gamblers (SOGS‐R). Mean age clustered around 70 years across severity groups, with no significant age differences. Males were more likely to score 3–4 on the SOGS‐R (*p* < .01), while marital status, education, employment, and residence did not differ significantly across groups. Those scoring ≥5 were most likely to be current smokers (*p* < .05). Problem and pathological gamblers engaged in a greater variety of gambling activities (*p* < .001), with slots and VLTs being the key differentiators at SOGS‐R ≥ 2 (*p* < .001); lottery participation did not differentiate severity groups. Bar/lounge VLT gambling was markedly more prevalent among higher‐severity gamblers (28.4% at SOGS‐R = 0 vs. 43–80% at SOGS‐R 1–5+, *p* < .05). While winning money and entertainment were universal motivations, passing time, escaping loneliness, and forgetting problems emerged as additional motivations among those scoring ≥3. Higher SOGS‐R scores were also associated with greater time spent gambling (>1 h/week) and higher monthly expenditure. SOGS‐R item analysis revealed that items related to borrowing money were rarely endorsed despite comprising nearly half the scale, suggesting standard problem gambling indicators may underperform in older adult populations.
‍Cousins S.O. & Witcher C.S., 2006^20^	Canada	Cross‐sectional	444	NA	Ad hoc structured interview; CATI	Among adults aged 65+, 266 had ever played bingo, of whom only 50 had played in the past 12 months. Active bingo players played 1–2 times per week on average, spending a mean of $20.96 per session. Compared to non‐players, bingo players showed higher overall gambling participation (92% vs. 78.2%, *p* < .05) and spent significantly more time gambling (10.32 vs. 3.11 h/month, *p* < .01). Bingo players were less likely to consume alcohol (80% drinking ≤1 drink/month vs. 55.6% of nonplayers, *p* < .01) and significantly less physically active (23% reporting no intention to exercise vs. 13.1% of nonplayers, *p* < .01), while smoking and eating habits did not differ significantly. Regression analyses identified key predictors of bingo expenditure: socially, women receiving income supplements, living in rental accommodation, with lower education and ≥1 health limitation explained 16% of variance (*p* < .001); in terms of lifestyle, non‐drinking was the only significant predictor, explaining 4%–5% of variance.
Potenza M. N. et al., 2006^21^	USA	Observational analysis of telephone screening Logistic regression analyses from CCPG helpline	1018	NA	Standardized telephone questionnaire from CCPG	Older adult problem gamblers (*N* = 168, 16.5% of total helpline callers, mean age 61.3 ± 5.9 years, range 55–78) were compared to younger adult callers (mean age 38.6 ± 9.3 years). Multivariate analyses distinguished the groups across eight of nine categories. Older adults reported lower income and longer gambling durations, with fewer types of problematic gambling. Slot machines were the predominant form of problematic gambling among older adults, who were less likely to report problems with table games. Psychiatrically, older adults showed similarly high rates of gambling‐related depression (74.4%) and suicidality (21.8%) compared to younger adults (80.5% and 26.0%, respectively), but were less likely to report gambling‐related anxiety. Gambling‐related financial problems were prevalent in both groups (74.0% vs. 82.6%), though older adults were less likely to report family problems, illegal behaviors, or arrests. Patterns of indebtedness were similar regarding credit sources (76.4% vs. 70.6%), but older adults were less likely to report debt to acquaintances, bookies, or loan sharks. Older adults also showed lower rates of daily tobacco use, current/past drug problems, and family history of drug abuse, while alcohol problems were comparable across groups (18.4% vs. 17.9%).
LaiD.W., 2006^22^	Canada	Cross‐sectional	2272	NA	Ad hoc structured questionnaire	Among 2257 older Chinese Canadians, 26.6% reported gambling, with mahjong being the predominant activity (75.3%), followed by slot machines (13.9%), cards (6.9%), and lottery (3.1%). Most gambled fewer than 4 times/month (45.1%) or 1–3 times/week (42.3%), primarily at others' homes (47%) or their own home (38%). Gamblers were more likely to be male, have elementary‐to‐secondary education, live in Winnipeg or Edmonton, have immigrated from Hong Kong or Southeast Asia, have lived longer in Canada, report higher social support and stronger Chinese ethnic identity, and face more barriers to healthcare. Hierarchical logistic regression confirmed these predictors: male gender (OR = 1.44), living in Winnipeg (OR = 3.77), Edmonton (OR = 2.22), Montreal (OR = 1.91), or Vancouver (OR = 1.53), longer Canadian residence (OR = 1.02/year), greater social support (OR = 1.08), stronger Chinese ethnic identity (OR = 1.11), and more service barriers (OR = 1.03) all increased gambling likelihood. Conversely, post‐secondary education (OR = 0.61) and higher life satisfaction (OR = 0.84) were protective factors.
Philippe F. & Vallerand R. J., 2007^23^	Canada	Cross‐sectional	810	NA	SOGS‐R; GPS	Among the total respondents aged 55+, 385 (47.5%) had gambled in the past 12 months, with a 12‐month prevalence of pathological gambling of 1.2% (SOGS‐R ≥ 5, *N* = 10) and 1.6% at‐risk (SOGS‐R 3–4, *N* = 13). SOGS‐R category did not differ by sex, age, education, or socioeconomic status. Obsessive passion was positively correlated with gambling severity (r*p* = .49, *p* < .01), while harmonious passion was negatively correlated (rp = −0.24, *p* < .01). ANOVA revealed a significant passion × SOGS‐R interaction (*F* = 13.66, *p* < .01): pathological gamblers showed significantly higher obsessive passion (M = 5.17) than both nonproblem (M = 1.79) and at‐risk gamblers (M = 2.25), and marginally lower harmonious passion (M = 2.50) compared to nonproblem (M = 3.95) and at‐risk gamblers (M = 4.13). Nonproblem and at‐risk gamblers did not differ on either passion type. Notably, a reversal pattern emerged: pathological gamblers scored higher on obsessive than harmonious passion, while the opposite held for non‐problem and at‐risk gamblers.
Clarke D. & Clarkson J., 2008^24^	New Zealand	Cross‐sectional	104	NA	GMS	Among older adult gamblers (*N* = 104), 64% gambled weekly or more, with 74% spending ≤NZ$10 per session. Most common activities were Lotto (71%), raffles (67%), and scratch tickets (62%), followed by casino EGMs (41%), races (39%), non‐casino EGMs (28%), and bingo (20%). Higher per‐session spending (> NZ$10) was significantly associated with casino EGMs (78% vs. 29%), non‐casino EGMs (59% vs. 17%), races (59% vs. 31%), and bingo (33% vs. 16%; all *p* < .05), but not with Lotto, raffles, or scratch tickets. Gender differences were minimal: men gambled more on races (49% vs. 32%, *p* < .05), while women more frequently played raffles (75% vs. 56%, *p* < .05) and bought scratch tickets weekly (33% vs. 15%, *p* < .05), with no significant differences in frequency, number of activities, or overall expenditure. Motivationally, external regulation (M = 8.18) and amotivation (M = 5.51) were the dominant motives across the sample, both significantly higher than identified regulation (M = 4.58; both *p* < .01), with no gender differences across any subscale. Regular continuous gamblers spent significantly more than noncontinuous gamblers (41% vs. 12% spending > NZ$10, *p* < .05) and scored higher on intrinsic motivation subscales (stimulation, knowledge, accomplishment) and introjected regulation and amotivation (all *p* < .05), suggesting a more intense motivational and expenditure profile.
‍‍Kerber C. S., Black D. W. & Buckwalter K., 2008^25^	USA	Cross‐sectional	40	NA	SOGS; GDS‐SF; NODS; PDQ‐4; MMSE; MINI.	The sample consisted of older adult recovering pathological gamblers (62.5% male, 95% Caucasian, mean age 62 ± 6.13 years, range 55–83), predominantly married (65%), with a mean education of 13.13 ± 1.6 years and normal cognitive functioning (MMSE mean = 29 ± 1.2). Gambling severity was moderate‐to‐severe (SOGS mean = 13.4 ± 3.53/20; NODS mean = 7.9 ± 2.25/10; *r* = .79, *p* = .01). Psychiatric comorbidity was substantial: only 1 participant had no Axis I or II diagnosis (mean Axis I diagnoses = 2.25 ± 1.4; mean Axis II = 1.9 ± 2.4). Mood disorders affected 82.5%, all with major depression, and 20% with bipolar episodes (GDS mean = 4 ± 4.05; 33% scoring ≥5 indicating current depression). Anxiety disorders were present in 47.5%, including panic disorder (27.5%) and generalized anxiety disorder (22.5%). Alcohol dependence was reported by 32.5%. Personality disorders were identified in 60% (PDQ‐4+ mean = 26.05 ± 12.59), with obsessive‐compulsive (37.5%), avoidant (27.5%), and depressive (25%) personality disorders being most prevalent.
Southwell J. et al., 2008^26^	Australia	Cross‐sectional	414	NA	CPGI; Self‐administered survey; Postal/web survey; Semi‐structured telephone interviews	Among older EGM players (*N* = 404), 40% spent less than 1 h per week on EGMs while 14% spent more than 5 h. Financial investment in EGMs was negatively associated with age (*p* = .016), but unrelated to income or employment. Those without a partner were twice as likely as partnered respondents to spend ≥5 h/week gambling (21% vs. 9%, *p* = .001). Primary motivations were club support (46%), winning money (45%), and socializing (41%), while boredom reduction (39%), isolation (34%), and stress/depression escape (33%) were also commonly reported. Women were more motivated by socializing (44% vs. 33%, *p* = .031) and stress escape (38% vs. 25%, *p* = .008). Those with disability, lower income (<$20,000), unemployment, and no partner showed significantly higher overall motivation scores and were more likely to gamble to reduce isolation and escape problems (all *p* < .05). Problem/moderate‐risk gamblers (identified via CPGI) were more likely to be male (*p* = .012), single (*p* = .012), casino gamblers (*p* = .022), to draw on savings (*p* < .001), hold erroneous beliefs about gambling outcomes, bet beyond their means, and report reduced session control; logistic regression confirmed younger age (60–69), male gender, single status, excitement‐seeking, and winning motivation as significant predictors. Regarding venue promotions, 50% had participated, with those aged 70+ more likely to do so (59% vs. 43%, *p* = .001); among participants, 18% spent more time and 14% more money due to promotions, with significantly greater impact among moderate‐risk/problem gamblers (more time: 33% vs. 14%, *p* = .009; more money: 27% vs. 11%, *p* = .020).
Zaranek R. R., & Lichtenberg P. A., 2008^27^	USA	Cross‐sectional	1410	NA	Lie/Bet Questionnaire; SF‐12; Questions about housing, health, transportation, and service utilization.	Among 1410 older adults (mean age 71.4 years, 70% female, 82.7% African American), 65.8% had annual incomes below $20,000, 40.8% had less than high school education, and 64% rated their health as good‐to‐excellent. Casino visitation was reported by 57.8%, with 17.2% visiting monthly or more. Problem gambling prevalence (Lie/Bet Questionnaire) was 10.4% overall, rising to 18.2% among casino visitors. Bivariate analyses identified significant associations between problem gambling and lower income (*r* = .08, *p* < .01), lower education (*r* = .07, *p* < .01), male gender (*r* = .12, *p* < .001), widowed status (*r* = −.05, *p* < .05), poorer mental health (*r* = .18, *p* < .001), poorer self‐rated health (*r* = .12, *p* < .001), lower senior optimism (*r* = .09, *p* < .001), lower social support (*r* = .09, *p* < .01), household transportation access (*r* = .05, *p* < .05), lower community involvement (*r* = .07, *p* < .01), and more frequent casino visits (*r* = .18, *p* < .001). Logistic regression confirmed casino visits as the strongest predictor (monthly+ visitors 2.6 times more likely to show problem gambling), followed by income below $20,000 (OR = 1.4), marital status, mental and physical health, senior optimism, transportation, and social support (all *p* < .01–.001). Age, gender, widowhood, community involvement, and social activities were not significant predictors in the full model.
Clarke D. & Clarkson J., 2009^28^	New Zealand	Cross‐sectional	104	NA	SOGS‐R; GHQ‐12; GMS	Among 104 older adults (median age 73, M = 74.59 ± 4.50, range 66–87; 63 women, 41 men; predominantly retired, widowed/single/divorced Caucasians), 64% gambled weekly or more, with 74% spending ≤NZ$10 per session. Problem gambling prevalence (SOGS‐R ≥ 3) was 9%, with no significant gender differences. No significant differences emerged between men and women on any variable. Problem gambling was significantly correlated with gambling frequency, number of activities, largest amount gambled, parental gambling history, and multiple motivational subscales (intrinsic motivation toward knowledge and stimulation, external/introjected/identified regulation, and amotivation), but not with psychological distress (*r* = −.03). Hierarchical regression showed situational variables accounted for 32% of SOGS‐R variance (*p* < .001), rising to 44% with motivational variables added (*p* < .05); significant individual predictors in the final model were gambling frequency, parental gambling, intrinsic motivation toward stimulation, and amotivation (suppressor effect; *F*(12,91) = 5.94, *p* < .001). Parental gambling explained the largest unique variance (9%), followed by frequency and amotivation (6% each) and stimulation motivation (5%). By activity type, EGM players (casino and non‐casino), bingo players, race bettors, scratch ticket players, and card game players all showed significantly higher problem gambling scores than non‐participants in those activities; no significant differences emerged for other casino games, Lotto, or raffles.
‍Ng V. C. N., 2011^29^	Singapore	Cross‐sectional	74	NA	Structured questionnaire	Among 74 older adults (82% female, 91% Chinese, mean age 71.62 ± 7.33 years, range 60–91), socioeconomic vulnerability was marked: 92% had primary education or lower, mean monthly income was $239 (SD = 129.42), and 69% depended on family allowances. Most (66%) lived in small flats (3‐room or smaller). Marital status was predominantly married (41%) or widowed (38%). Past‐month gambling was reported by 27% (*n* = 20), with 4D as the most popular activity (75%), followed by Toto (20%) and mahjong (5%), at an average frequency of ~2 times/week and mean weekly expenditure of $11.88 (SD = 13.76). Mean gambling onset age was 34.65 years (SD = 16.47), with 55% having started by age 35; gambling was introduced primarily by friends or fellow gamblers (95%). Perceptions of gambling were mixed: 33% viewed it as a vice, 55% as acceptable recreation, and 26% as a means to win money. Gamblers reported significantly more discretionary time than nongamblers (64 vs. 38 h/week, *t* = 2.581, *p* < .05). No significant associations were found between gambling and gender, race, marital status, education, housing type, or income. Notably, 97% of respondents were unaware of where to seek help for gambling problems.
‍Martin F., Lichtenberg P. A. & Templin T. N., 2011^30^	USA	Secondary Analysis of a Longitudinal study	247	NA	Structured questionnaire	The follow‐up sample (*N* = 247, 89% female, predominantly widowed [43%] and African American [91%], mean age range 60–85+) showed that education and income were the only sociodemographic factors significantly associated with casino visit frequency (both *X* ^2^ = 15.54, *p* = .004), with higher education and income predicting more frequent visits. Gambling frequency declined significantly over time: monthly‐or‐more gambling dropped from 25.5% to 16.9%, while nongambling in the past year increased from 32.9% to 48.6% (*p* < .001). Attitudes toward casinos remained generally positive, with perceptions of safety increasing (75.3% to 82.3%, *p* < .05), while perception of casinos as good for Detroit decreased slightly (66.7%–59.8%, *p* < .005). Primary gambling motivations were entertainment (79.5%) and winning money (62.7%), with additional intrinsic motivations including alleviating boredom/loneliness (38.2%) and escaping grief (22.2%).
Pilver C. E., Libby D. J., Hoff R. A., & Potenza M. N., 2013^31^	USA	Secondary analysis (data from NESARC). Prospective and longitudinal epidemiological study.	10231	NA	AUDADIS‐IV; SF‐12	At Wave 1 (*N* = 10,231), 67.3% were low‐frequency/nongamblers (LFG/NG), 29.9% low‐risk gamblers (LRG), and 2.8% at‐risk/problem gamblers (ARPG), of whom 84.7% endorsed 1–2 problem gambling features, 13.3% endorsed 3–4, and 2.0% endorsed ≥5. Baseline ARPG was associated with age, gender, marital status, education, employment, income, mood/anxiety/substance‐use disorders, tobacco and alcohol use, stressful life events, and physical health (all *p* < .02). Longitudinally, ARPG was associated with elevated incidence of any Axis‐I disorder (OR = 2.09, *p* = .033) after sociodemographic adjustment, though this became nonsignificant with full covariate adjustment. For mood disorders, ARPG was initially associated with incident mania (OR = 3.43, *p* = .015), but this did not survive covariate adjustment; LRG was negatively associated with incident hypomania (OR = 0.33, *p* = .017). For anxiety disorders, ARPG remained independently associated with incident generalized anxiety disorder across all models (OR = 2.51, *p* = .011), while the association with panic disorder did not survive adjustment. For substance use, ARPG was independently associated with incident any substance‐use disorder in the fully adjusted model (OR = 2.61, *p* = .004), while associations with nicotine dependence and alcohol abuse/dependence became non‐significant after full adjustment.
Tse S. et al., 2013^32^	Singapore	Cross‐sectional	3010	NA	Structured questionnaire Face‐to‐face interview; PGSI	Lifetime gambling participation was 56.2%, with 69.7% of lifetime gamblers having gambled in the past 12 months (population prevalence 39.2%). Of past‐year gamblers, 69.9% were no‐risk, 15.5% low‐risk, 12.4% moderate‐risk, and 2.2% problem gamblers (PGSI). Over 75% of past‐year gamblers were Chinese, married, and Buddhist/Hindu/Taoist. Problem gamblers reported younger gambling onset, higher monthly gambling expenditure, more time spent gambling, and greater involvement in slot machines and card/board games, while only 0.4% of no‐risk gamblers had sought any intervention. Logistic regression identified five independent predictors of problem gambling: older age group, younger gambling onset, higher monthly personal income, higher total gambling expenditure, and no help‐seeking experience; conversely, stocks/options/commodities trading and Singapore lottery (4D) participation were associated with lower odds of problem gambling (all *p* < .001).
‍Botterill E. et al., 2015^33^	Australia	Cross‐sectional	183	NA	PGSI; Revised UCLA Loneliness Scale	Most older adult gamblers were partnered, not employed, and preferred venue‐based gambling; lotteries and scratch cards were the most popular activities, followed by EGMs, bingo, horse racing, and raffles. Over 13% were moderate‐to‐high‐risk problem gamblers, with moderate‐risk gamblers predominantly male and high‐risk predominantly female. Un‐partnered older adults showed significantly higher problem gambling levels than partnered adults (*F*(1,179) = 10.4, *p* = .001). Loneliness was higher among un‐partnered men than partnered men, but did not differ significantly by marital status among women (gender × marital status interaction: *F*(1,179) = 6, *p* = .015). Mediation analysis confirmed that loneliness mediated the relationship between marital status and problem gambling, with un‐partnered status associated with greater loneliness, which in turn predicted higher problem gambling. Moderated mediation revealed that gender moderated the marital status–loneliness pathway but not the loneliness–problem gambling pathway: the mediation model was supported for men (un‐partnered → lonelier → more problem gambling) but not for women, for whom marital status did not significantly predict loneliness.
‍Medeiros G. C. et al., 2015^34^	Brazil & USA	Cross‐sectional and cross‐cultural comparative	170	NA	SCI‐PG‐DSM‐5; GSAS; MINI; MIDI	Brazilian (BR, *n* = 89) and US (*n* = 81) older adult gamblers (total *N* = 170; 67 men, 103 women; mean age 64.42 ± 3.86 years) showed largely similar sociodemographic profiles, differing only in marital status (BR more likely partnered). BR gamblers had a later gambling onset, later onset of gambling disorder and lower urge scores, though overall gambling severity did not differ. BR gamblers showed higher rates of lifetime major depressive disorder, while US gamblers showed a trend toward higher lifetime alcohol‐related problems and greater lifetime history of risky behaviors (sexual, spending, stealing). Binary logistic regression identified urge intensity and age of gambling disorder onset as the key variables differentiating the two groups.
Sauvaget A. et al., 2015^35^	Spain	Case report	1	NA	MINI 5.00; SOGS; DSM‐IV Diagnostic Questionnaire of Stinchfield; TCI‐R; BIS‐11; AUDIT; UPPS‐P; SCL‐90‐R	No signs of cognitive impairment or neurodegenerative diseases were found following neuropsychological evaluation. However, he had several risk factors for gambling disorder, including adjustment difficulties, stressful events in his life, a previous gambling addiction in physical casinos at the age of 50 and dysfunctional personality traits. The switch to online gambling may have been influenced by his isolation, mobility difficulties and his high level of education, which made it easier for him to access the Internet.
‍Black D. W. et al., 2017^36^	USA	Prospective follow‐up study and Longitudinal study	175	NA	SOGS; NODS; MIDI; SCID; MINI; SPID‐IV; BDI; BIS; Family History Research Diagnostic Criteria; BIS; MOS; GSAS; TLFB.	The sample comprised older PGs (*n* = 53, mean age 66.5 ± 6.8 years, 64% female, 94% Caucasian, 57% divorced), younger PGs (*n* = 72, mean age 27.4 ± 7.7 years), and elders without PG (*n* = 50, mean age 71.2 years), followed for a mean of 2.6 ± 1.4 years (range 0–4.6). Older PGs differed from elders without PG in being younger, more likely divorced, less educated, more impulsive, more depressed, and showing higher rates of psychiatric hospitalization, childhood maltreatment, suicide attempts, and lifetime comorbidities including alcohol use disorder (53% vs. 18%), mood disorders (58% vs. 34%), and anxiety disorders (42% vs. 20%). Compared to younger PGs, older PGs were more likely female, Caucasian, and divorced, preferred slots (66% vs. strategic games 74%), had later PG onset (50.0 ± 13.2 vs. 22.1 ± 7.3 years), and showed lower rates of drug use disorders (6% vs. 42%) and ADHD (8% vs. 24%). No significant differences in PG outcomes were found between older and younger PGs: older PGs spent 49.3% of follow‐up in chronic PG, 19.9% in partial remission, and 21.9% in remission, compared to 45.3%, 16.6%, and 28.5%, respectively, for younger PGs. Odds of problem gambling decreased by 0.65 per year for both groups, while odds of remission/partial remission increased (younger PGs OR = 2.26; older PGs OR = 1.99). Time to relapse did not differ between groups (*p* = .833), and no controls developed PG during follow‐up.
Van der Maas M., Mann R.E. et al., 2017^37^	Canada	Cross‐sectional	1978	NA	PGSI; Behavioral questionnaire; Tablet‐assisted personal interviews:	Bus‐tour patronage (past‐year) was reported by 41% of the sample and was significantly more common among women, foreign‐born participants, retirees, and older age groups; marital status did not vary significantly. Notably, 67% of Asian and 76% of East Indian participants reported past‐year bus‐tour patronage (both *p* < .001). Bus‐tour patrons showed significantly higher rates of monthly‐or‐more slot machine play, higher gambling venue visit frequency, lower per‐visit spending, and were more likely to be classified as problem gamblers (PGSI). In mixed‐effects logistic regression, bus‐tour patronage was not significant in the unadjusted model, but emerged as a significant predictor of problem gambling after demographic adjustment (OR = 1.71). Additional significant predictors included past‐month slot machine participation (OR = 5.48), per‐visit spending (approximately doubled odds), separated/divorced status (OR = 2.72), and nonnative Canadian origin (OR = 2.46), while older age was associated with lower odds of problem gambling. Female gender and retirement status were not significant predictors.
Turner N. E. et al., 2018^38^	Canada	Cross‐sectional	2103	NA	PGSI; ad hoc questions about SDQ, GAQ, FA, EGS, DC, GVS	The participants engaged in 3.6 forms of gambling in the past year, and 78.6% reported playing slot machines or other forms of electronic gaming machines monthly or more often. They reported spending an average of 3.29 h gambling at casinos or racinos per visit and 134.9 h at casinos or racinos per year. Just over one‐fifth of the sample reported spending more than $6000 in casinos or racinos in the past year. Based on the PGSI, the proportion of the sample experiencing severe problem gambling (PGSI = 8+) was 6.9%, and an additional 20.3% reported moderate gambling problems (PGSI = 3–7).
Parke A., Griffith M., Pattinson J. & Keatley D., 2018^39^	UK	Cross‐sectional	595	NA	Clock Drawing Test; PGSI; Canadian Study of Health and Aging Clinical Frailty Scale; GPM SF‐12; De Jong Gierveld Loneliness Scale; GDS‐15; GAI	Problem gambling was present in 16% of participants (8.4% mild, 4.1% moderate, 3.5% severe). Anxiety symptoms affected 63.7% (38.2% mild, 25.5% clinical), mild‐to‐severe depression was present in 24.7%, loneliness affected 66.6%, and 64.5% reported physical pain (15.8% mild, 24.2% moderate, 24.5% severe), with 36.3% experiencing at least moderate physical frailty. No significant gender differences emerged on any variable; frailty (*r* = .44, *p* < .001) and pain (*r* = .22, *p* < .001) increased with age, while loneliness, depression, anxiety, and problem gambling did not. Path analysis yielded a well‐fitting final model (*χ* ^2^(4) = 7.39, *p* = .117, RMSEA = 0.038, CFI = 0.996) in which loneliness (*β* = .24, *p* < .001) and anxiety (*β* = .23, *p* < .001) were the only direct predictors of problem gambling. Clinical frailty and geriatric pain had small but significant indirect effects on problem gambling mediated through depression and anxiety, and depression's effect on problem gambling was fully mediated by anxiety. Direct effects of frailty on anxiety and problem gambling, and of geriatric pain and depression on problem gambling, were non‐significant and removed from the final model.
Granero R. et al., 2020^40^	Spain	Cross‐sectional	361	NA	Diagnostic Questionnaire for Pathological Gambling according to DSM Criteria; SOGS; SCL‐90‐R; self‐report questionnaire for identifying lifetime exposure to traumatic events; semi‐structured interview for sociodemographic features	The prevalence of GD in the study was 1.4%. Two clusters were identified: (a) cluster 1 (labeled as “low risk of gambling problems”, *n* = 265, 73.4%), which included the higher proportion of nongamblers or individuals who engage only in non‐strategic gambling, women, widowed, and lower levels of education (no individual into this group met criteria for GD); and (b) cluster 2 (labeled as “higher risk of gambling problems,” *n* = 96, 26.6%), which included the higher proportion of men, who reported both nonstrategic and strategic gambling preferences (all participants diagnosed of GD were grouped into this cluster), older age, longer history of gambling, higher gambling severity, higher use of substances and worse psychopathological state. The elderly constitute a heterogeneous group with regard to gambling phenotypes.
Venuleo C. et al., 2021^41^	Italy	Cross‐sectional	165	NA	PGSI; MSPSS; DJGLS; FS	11.5% of the sample were problem gamblers and 26.7% were moderate‐risk gamblers (with 11.5% low‐risk and 50.3% non‐problem gamblers). Scratch cards were the most common gambling activity (47%), followed by Lotto (26.2%). Problem gamblers were more likely to play slot machines (5.5%) and to gamble alone (41%). The main motivations were making money (65.5%) and distraction from personal problems (50.9%). 50.9% of participants expressed reluctance to seek help, primarily due to cultural beliefs and fear of judgment. Problem gamblers showed higher social loneliness, lower perceived social support, and lower overall well‐being compared to nongamblers (*p* < .001), but no significant differences in emotional loneliness were observed. Moderate‐risk gamblers were slightly younger (mean age: 65.09 years) than nongamblers (mean age: 68.27 years).
del Pino‐GutiérrezA. et al., 2021^42^	Spain	Cross‐sectional	361	NA	DQPG; SOGS; SCL‐90 R; LE	The percentage of participants who reported an absence of gambling activities was 35.5%, while 46.0% reported only nonstrategic gambling, 2.2% only strategic gambling and 16.3% both nonstrategic plus strategic gambling. Gambling form with highest prevalence was lotteries (60.4%), followed by pools (13.9%) and bingo (11.9%). The prevalence of gambling disorder was 1.4%, and 8.0% of participants were at a problematic gambling level. Onset of gambling activities was younger for men, and male participants also reached a higher mean for the bets per gambling‐episode and the number of total gambling activities. Risk factors for gambling severity in the sample were not being born in Spain and a higher number of cumulative lifetime life events, and gambling severity was associated with a higher prevalence of tobacco and alcohol abuse and with worse psychopathological state.
Zhang, N. et al., 2022^43^	USA	Cross‐sectional	3157	NA	MMSE; SDMT; EBMT; DB; MSOGS; PGSI; ADL	Higher gambling participation was significantly associated with higher global cognition (*b* = 0.008, SE = 0.003, *p* < .05), executive function (*b* = 0.118, SE = 0.049, *p* < .05), and episodic memory (*b* = 0.009, SE = 0.004, *p* < .05). Higher problem gambling was significantly related to lower Mini‐Mental State Examination (*b* = −0.105, SE = 0.031, *p* < .001). Guiding older adults' gambling behaviors and intervening in problem gambling timely might be crucial for their cognitive function.
Gorenko J.A. & Konnert C.A., 2023^44^	Canada	Secondary analysis from longitudinal study	571	NA	PGSI	Results supported unidimensionality of the PGSI and item polarity (rpm range = 0.42–0.85). Infit and outfit statistics showed mixed model misfit for three items. The use of three response options were deemed productive for measurement, but almost always was not frequently endorsed. Model‐person separation (1.00) and reliability (0.50) were poor, and model‐item separation (6.12) and reliability (0.97) were excellent. There was no significant DIF between older adults by gender. Possible modifications to improve the PGSI for use with older adults are discussed.
Gorenko, J.A., 2025^45^	Canada	Secondary analysis from longitudinal study	670	NA	PGSI; Gambling Frequency 10 items; LEQ; PAI; CIDI‐SF; physical health status (1 item)	The final model showed significant effects (*p* < 01) for linear time (PGSI scores declined across waves; *β* = − 06); gender (women's mean PGSI greater than men's; *γ* = 26); social support (less support associated with greater PGSI; *γ* = 05); and an interaction between age and stressful life events (effect of stressful events on PGSI is lesser with greater age; *γ* = − 02). Simple slopes (at mean age of 66 at wave 1, and ±1 SD) showed that at 61 (*γ* = 35) and 66 years (*γ* = 23), mean PGSI scores were greater with each additional stressful life event; there was no significant effect for stressful events at age 71. These findings suggest women, those with less social support, and greater stressful life events may be at greater risk of problem gambling, while greater age weakens the effect of stressful events and problem gambling.

Abbreviations: ADL, Activities of Daily Living; ASI, Addiction severity index; ARPG, At‐Risk/Problem Gamblers; AUDADIS‐IV, Alcohol Use Disorder and Associated Disabilities Interview Schedule—Diagnostic and Statistical Manual of Mental Disorders—Fourth Edition; AUDIT, Alcohol Use Disorders Identification Test; BIS‐11, the Barratt Impulsivity Scale; CATI, Computer‐assisted telephone survey; CCPG, Connecticut Council on Problem Gambling; CES‐D, Center for Epidemiologic Studies Depression scale; CIASOA, Chinese Internet Addiction Scale for Older Adults; CIDI‐SF, Composite International Diagnostic Interview short‐form; DB, Digit Span Backward; DC, Distance from Casino; DJGLS, De Jong‐Gierveld Loneliness Scale; DQPG, Diagnostic Questionnaire for Pathological Gambling; EBMT, East Boston Memory Test‐Immediate Recall; EGM, electronic gaming machine; EGS, Expenditure in Gambling Survey; FA, Frequency of Attendance; FS, Flourishing Scale; GAQ, Gambling Activities Questionnaire; GDS‐15, Geriatric Depression Scale Short Form; GHQ‐12, General Health Questionnaire GMS, Gambling Motivation Scale; GVS, Group Visit Survey; LE, Life events; LEQ, Life Events Questionnaire; LRG, Low‐Risk Gamblers; MINI 5.00, Mini International Neuropsychiatric Interview; MMSE, Mini‐Mental State Examination; MSOGS, Modified South Oaks Gambling Screen; MSPSS, Multidimensional Scale of Perceived Social Support; NIMHS DIS, Diagnostic Interview Schedule; NODS, National Opinion Research Center DSM‐IV Screen; PAI, Personality Assessment Inventory; PDQ‐4, Personality Disorder Questionnaire; PGs, Pathological gamblers; PGSI, Problem Gambling Severity Index; SCL‐90 R, Symptom Checklist–Revised; SDMT, Symbol Digit Modalities Test; SDQ, Sociodemographic Questions; SF‐12, The 12‐item Short Form Health Survey; SF‐36, Short Form Health Survey 36; SOGS, South Oaks Gambling Severity Screen; SOGS‐R, Revised South Oaks Gambling Screen; SWLS, Satisfaction with Life Scale; TCI‐R, Temperament and Character Inventory–Revised; ULS‐8, Loneliness Scale; UPPS‐P, Impulsive Behavior Scale.

### Study risk of bias assessment

In this systematic review, rigorous tools were employed to meticulously assess the Risk of Bias. Specifically, the Newcastle–Ottawa Quality Assessment Scale was utilized for Cross‐Sectional, Observational Cohort and Comparative Observational Studies (NOS); while the JBI Critical Appraisal Checklist for Case Reports was used for a case report.

## RESULTS

### Study selection

The articles resulting from the research phase in the databases listed above produced a total of 40,263 reports. The duplicate manuscripts were deleted until a number of 35,116 unique reports were produced. These unique reports were examined by the title and abstract and 203 were selected. These reports were opened and analyzed in full‐text version. In conclusion following full‐text screening 35 studies were included in the systematic review. The PRISMA flow diagram in Figure 1 outlines the selection process in detail.

**Figure 1:**
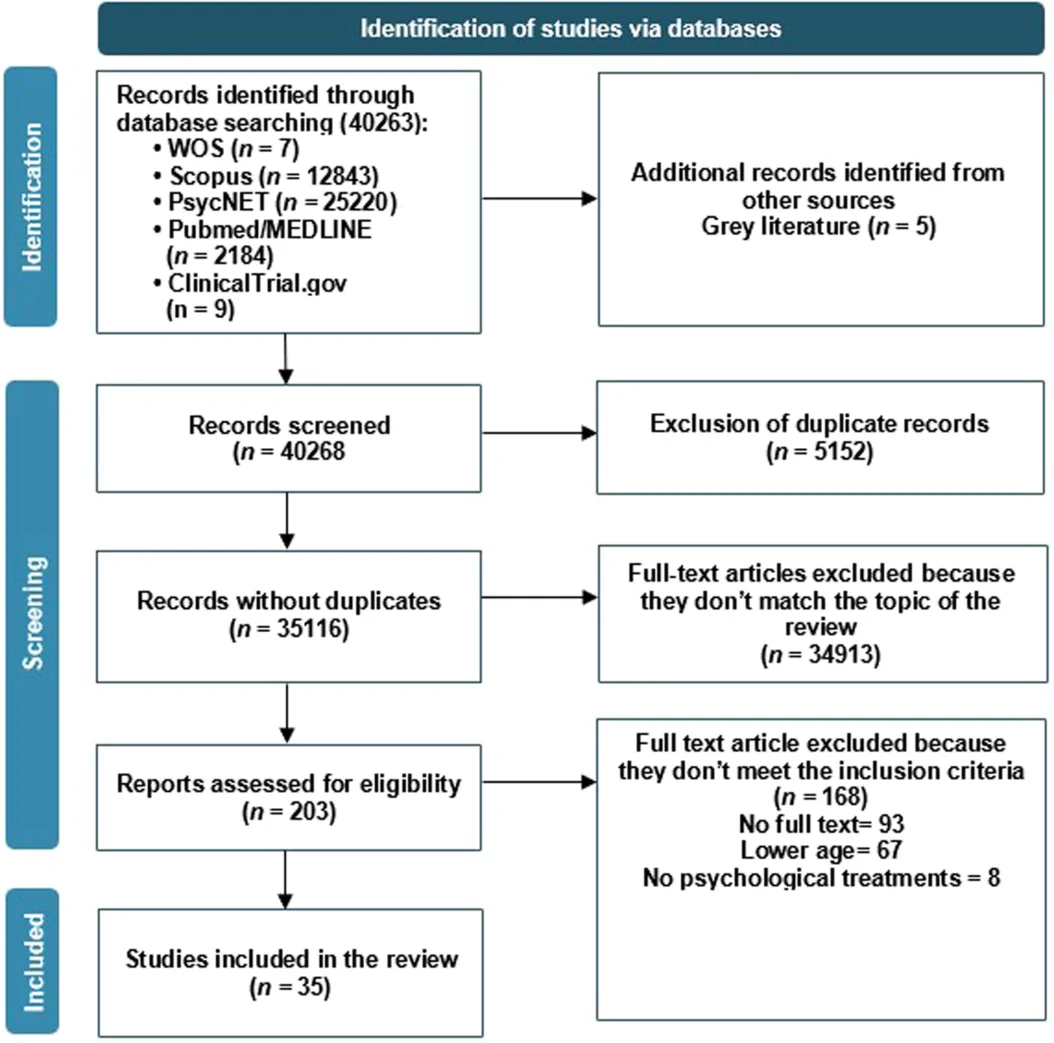
PRISMA flow diagram.

As shown in the flow diagram, several studies initially appeared to meet the inclusion criteria but were ultimately excluded for specific reasons: a total of 168 studies did not meet the inclusion criteria: 93 studies were excluded due to the unavailability of full text, 67 studies involved participants younger than the required age threshold (below 55 years), and 8 studies did not focus on psychological or psychotherapeutic interventions.

### Study characteristics

The 35 included studies (Table 1) were published between 2000 and 2025 and were conducted mainly in North America and Europe. Study designs included primarily cross‐sectional studies, as well as longitudinal studies, secondary analyses, observational studies, comparative studies, and one case report. Sample sizes ranged from small case reports (*n* = 1) to large population‐based studies exceeding 10,000 participants. All studies examined gambling disorder among older adults, focusing on prevalence, severity, psychosocial factors, comorbidities, and associated risk factors. Outcome measures were heterogeneous and included assessments of gambling severity, psychological symptoms, cognition, quality of life, and social functioning.

### Risk of bias in studies

The results of the risk of bias assessment are presented in Tables 2 and 3. Overall, the cross‐sectional studies demonstrated good to very good methodological quality, with most scoring between 7 and 10 points on the adapted Newcastle–Ottawa Scale. Several studies achieved very high ratings (≥9 points), reflecting strong representativeness, use of validated assessment tools, and appropriate statistical analyses.^16^, ^18^, ^19^, ^26^, ^27^, ^31^, ^32^ A smaller group of studies, such as Cousins and Witcher and Botterill et al., received satisfactory ratings (5–6 points) due to limitations in sampling procedures, incomplete reporting of non‑respondents, or reliance on ad hoc measures.^20^, ^33^ For case reports, the JBI Critical Appraisal Checklist was applied. The only included case report, Sauvaget et al., met nearly all methodological criteria except for the reporting of adverse events, and was therefore judged as “Include” with minor concerns.^35^

**Table 2:** Newcastle—Ottawa Quality Assessment Scale for Cross‐Sectional Studies.

No.	First author, year	Selection				Comparability	Outcome		Total
		1	2	3	4		1	2	
1.	McNeilly & Burke, 2000	*	*	*	**	*	**	*	9
2.	Bazargan M. et al., 2001	*	*		**	**	*	*	8
3.	Ladd G. T. et al., 2003	*		*	**	*	**	*	8
4.	Burge A. N., 2004	*	*		**	**	*	*	8
5.	Desai et al., 2004	*	*	*	**	**	**	*	10
6.	Kaush, 2004		*		**	*	**	*	7
7.	Vander Bilt, J.V. et al., 2004	*	*		**	*	*	*	7
8.	Erickson et al., 2005	*	*	*	**	*	**	*	9
9.	Wiebe & Cox, 2005	*	*	*	**	*	**	*	9
10.	Cousins & Witcher, 2006		*	*		*	*	*	4
11.	Lai D.W., 2006	*	*		*	**		*	6
12.	Potenza et al., 2006		*		*	*	*	*	5
13.	Philippe & Vallerand, 2007	*	*	*	**	**	**	*	10
14.	Clarke D. & Clarkson J., 2008	*		*	**	**	**	*	9
15.	Kerber, Black & Buckwalter, 2008				**		**	*	5
16.	Zaranek & Lichtenberg, 2008	*	*	*	**	**	**	*	10
17.	Clarke D. & Clarkson J., 2009			*	*	*	**	*	5
18.	Southwell J. et al., 2008	*	*		**	**	**	*	9
19.	Martin, Lichtenberg & Templin, 2011	*	*	*	*	*	*	*	7
20.	Vincent C. K. Ng, 2011		*	*	*		*	*	5
21.	Pilver et al., 2013	*	*	*	**	**	**	*	10
22.	Tse et al., 2013	*	*	*	**	**	**	*	10
23.	Botterill et al., 2015		*		**	*	**	*	7
24.	Medeiros et al., 2015		*		**	**	**	*	8
25.	Black et al., 2017		*		**	*	**	*	7
26.	Van der Maas, Mann et al., 2017	*	*	*	**	**	**	*	10
27.	Parke, Griffiths, Pattinson & Keatley, 2018		*		*	*	*	*	5
28.	Turner N. E. et al., 2018	*	*	*	**	**	**	*	10
29.	Granero R. et al., 2020	*	*		**	**	**	*	9
30.	Venuleo C. et al., 2021	*	*		**	**	**	*	9
31.	del Pino‐Gutiérrez A. et al., 2021				**	**	**	*	7
32.	Gorenko J.A. & Konnert C.A., 2023	*	*		**	**	**	*	9
33.	Zhang N. et al., 2022	*	*		*	*	**	*	7
34.	Gorenko J.A., 2025	*	*		**	**	**	*	9

**Table 3:** JBI critical appraisal checklist for case reports.

No.	First author, year	D1	D2	D3	D4	D5	D6	D7	D8	Overall
1.	Sauvaget A. et al., 2015	Yes	Yes	Yes	Yes	Yes	Yes	No	Yes	Include

Domains

D1: Were patient's demographic characteristics clearly described?

D2: Was the patient's history clearly described and presented as a timeline?

D3: Was the current clinical condition of the patient on presentation clearly described?

D4: Were diagnostic tests or assessment methods and the results clearly described?

D5: Was the intervention(s) or treatment procedure(s) clearly described?

D6: Was the post‐intervention clinical condition clearly described?

D7: Were adverse events (harms) or unanticipated events identified and described?

D8: Does the case report provide takeaway lessons?

### Results of studies

The 35 studies identified meeting the inclusion criteria and providing a comprehensive, multi‐perspective overview of gambling behaviors, clinical correlates, and trajectories in older adults. The vast majority of these investigations (*n* = 28) utilized cross‐sectional designs, which precludes the establishment of temporal precedence or causal inference. Furthermore, the included literature exhibited substantial heterogeneity in how gambling behaviors were operationalized, spanning a wide spectrum from recreational participation to at‐risk and clinically significant gambling disorders.

#### Prevalence

Prevalence estimates for problem and pathological gambling varied markedly across studies, reflecting the diversity of assessment instruments and sampling strategies employed. Severe problem gambling was reported at 6.9% by Turner et al., while Clarke and Clarkson reported a 9% prevalence using the SOGS‐R.^28^, ^38^ Venuleo et al. observed a problem gambling prevalence of 11.5%, with an additional 26.7% of the sample classified as moderate‐risk.^41^ In Canadian community samples, Wiebe and Cox found that 1.6% of participants were problem gamblers and 1.2% were probable pathological gamblers.^19^ Similarly, Ladd et al. noted rates of 5.9% for problem gambling and 4.7% for pathological gambling, whereas Erickson et al. reported 6.4% and 3.8%, respectively.^13^, ^18^ A higher prevalence of 16% was identified by Parke et al. within a UK sample.^39^ Finally, regarding past‐month behaviors in specific cohorts, Ng revealed that 27% of low‐income Singaporean older adults had engaged in gambling activities.^29^

#### Sociodemographic and risk factors

Sociodemographic analyses consistently identified male gender as a significant predictor for being classified as a problem gambler.^26^, ^40^, ^42^ However, Gorenko found that women exhibited higher mean PGSI scores, demonstrating that female gender, lower social support, and an accumulation of stressful life events were robust predictors of greater gambling severity.^45^ Age‐stratified observations indicated internal variability within the geriatric cohort: younger segments of the older adult population (the “young‐old”) frequently exhibited higher gambling frequency and greater vulnerability to life stressors compared to older‐old subgroups.^13^, ^38^ Additional independent predictors of elevated problem gambling risk included lower income, being unmarried, and separated or divorced status. Environmental and contextual factors, such as participation in casino bus tours, were also positively associated with gambling.^13^, ^37^, ^38^ Regarding specific gambling formats, slot machines and electronic gaming machines (EGMs) emerged as the predominant problematic form of gambling across multiple samples.^15^, ^21^, ^36^, ^37^ Other formats included bingo, which correlated with lower education levels and physical, as well as mahjong and various lotteries, which were strongly linked to lower life satisfaction or specific ethnic identity affiliations.^20^, ^22^, ^29^, ^32^, ^43^

#### Motivations

Older adults endorsed a combination of monetary, social, and emotional motivations for gambling. McNeilly and Burke documented that primary drivers among casino patrons included relaxation/fun (68.1%), getting away for the day (43.7%), passing time (42.7%), and relieving boredom (30%). Similarly, Martin et al. (2011) identified entertainment (79.5%) and winning money (62.7%) as primary motivations, alongside boredom and loneliness relief (38.2%).^11^ Problem gamblers were significantly more likely to report gambling alone, and regular gamblers scored higher on dimensions of curiosity and escape.^26^, ^28^, ^42^ At a structural motivational level, Philippe and Vallerand found that obsessive passion was positively associated with gambling severity, whereas harmonious passion was negatively associated.^23^ Furthermore, path modeling by Parke et al. identified loneliness and anxiety as the only direct predictors of problem gambling, with frailty and pain exerting indirect effects through depression and anxiety.^39^ Loneliness was also found to completely mediate the relationship between marital status and problem gambling specifically among older men.^33^

#### Cognitive correlates

Empirical evidence regarding cognitive correlates in older gamblers yielded mixed outcomes. Zhang et al. found that older recreational gamblers demonstrated better global cognition and episodic memory compared to nongamblers, but exhibited significantly lower performance in executive function and working memory.^43^ In a clinical case study, Sauvaget et al. documented an older adult who developed severe online gambling disorder and accumulated massive debts despite exhibiting no global cognitive impairment, with the presentation instead characterized by severe social isolation and specific pre‐existing personality traits.^35^

#### Comorbidity

Psychiatric comorbidity was highly prevalent, particularly within clinical, helpline, and treatment‐seeking samples. Kerber et al. reported that among recovering pathological gamblers, 82.5% presented with mood disorders (all meeting criteria for major depression), 47.5% with anxiety disorders, and 60% with personality disorders (predominantly obsessive‐compulsive personality disorder at 37.5%).^25^ Potenza et al. noted that among older adults contacting a helpline, gambling‐related depression was endorsed by 74.4% and suicidality by 21.8%.^21^

Early‐onset gamblers exhibited significantly worse physical and psychological health outcomes compared to late‐onset gamblers.^14^ Phenotypic cluster analyses by Granero et al. identified a distinct high‐risk subgroup characterized by strategic gambling preferences, comorbid substance use, and poor overall psychological functioning.^40^ Bazargan et al. found that heavy/pathological gamblers reported significantly more stressful life events alongside lower psychological well‐being, higher anxiety, and more obsessive‐compulsive symptoms.^12^ Longitudinally, Pilver et al. demonstrated that at‐risk and problem gambling was independently associated with the subsequent development of incident generalized anxiety disorder (OR = 2.51) and any substance‐use disorder (OR = 2.61).^31^

#### Longitudinal trajectories

Longitudinal data indicated that while gambling severity can fluctuate or decrease over time, it remains heavily anchored to stable individual characteristics. Gorenko found that PGSI scores declined only modestly over five assessment waves, with most of the variance attributable to stable individual traits rather than time‐based changes.^45^ Black et al. observed no significant differences in problem gambling outcomes between older and younger pathological gamblers over a mean follow‐up of 2.6 years, with the odds of problem gambling decreasing by a factor of 0.65 per year for both age groups.^36^ Lastly, Kausch found that among treatment‐seeking older adult gamblers, over 25% had developed a gambling problem within 5 years, and nearly 50% within 10 years prior to admission.^15^

## DISCUSSION AND CONCLUSIONS

This systematic review provides an overview of Gambling Disorder and problematic gambling behaviors among adults aged 55 years and older, focusing on prevalence, associated clinical correlates, and available psychological interventions. The findings indicate that gambling‐related problems represent an important but still insufficiently characterized clinical issue in later life. The current literature does not support a qualitatively distinct disorder in older adults; instead, common vulnerability mechanisms appear to interact with age‐related circumstances, influencing the onset, maintenance, and clinical expression of gambling problems. The first objective of this review was to characterize the prevalence and severity of gambling‐related problems in older adults. The included studies showed highly variable prevalence estimates, ranging from low levels of problem gambling to substantially higher rates in selected samples. This variability reflects differences in assessment tools, recruitment strategies, and definitions of gambling‐related impairment rather than providing a stable epidemiological estimate. In particular, the frequent use of screening instruments rather than DSM‐based diagnostic evaluations limits direct comparison between studies; prevalence findings should therefore be interpreted cautiously, and future epidemiological studies should prioritize standardized diagnostic approaches adapted to older populations.^2^ A relevant implication emerging from these findings is that gambling problems in older adults should not be interpreted only through the lens of aging‐related difficulties. Although loneliness, financial strain, reduced social support, and stressful life events were frequently associated with gambling severity, these factors are also recognized correlates of gambling problems across age groups.^2^ The distinctive feature of later life is instead the accumulation of multiple transitions, retirement, changes in social roles, bereavement, reduced opportunities for alternative reinforcement, so that aging functions less as an independent risk factor and more as a context that modifies existing vulnerabilities. Consistent with this interpretation, the motivational findings suggest that gambling in older adults occupies a complex position between recreational activity and maladaptive coping strategy. Several studies reported that older adults gamble for entertainment, social interaction, relaxation, and boredom reduction.^11^, ^24^, ^28^ These motivations alone do not indicate psychopathology. However, when gambling becomes a primary mechanism for regulating distress, compensating for social isolation, or escaping negative emotional states, it may contribute to clinically significant impairment. This distinction has important clinical implications, as assessment should consider not only gambling frequency or financial consequences but also the psychological function that gambling serves. The findings also highlight substantial heterogeneity among older adults who experience gambling‐related problems. Some individuals appear to present gambling behaviors primarily associated with emotional vulnerability, while others show earlier gambling initiation, greater persistence, or broader psychiatric difficulties.^9^, ^40^ This variability supports the use of individualized clinical formulations rather than assuming a single pathway to Gambling Disorder in later life. An additional contribution of this review is the identification of biological and medical factors as an underexplored area in the current literature. Although most included studies focused on psychosocial correlates, clinicians should consider the possible contribution of neurological conditions, age‐related changes in reward processing, and medication exposure. In particular, dopamine agonists prescribed for Parkinson's disease and restless leg syndrome have been associated with impulse control disorders, including pathological gambling.^5^ Although not the focus of this review, medication‐induced gambling warrants systematic assessment in future studies and clinical evaluations of older adults. Psychiatric comorbidity represented another consistent finding across the included studies. Older adults with gambling problems, particularly those recruited from clinical or support settings, showed elevated levels of depression, anxiety, and suicidality.^21^, ^25^ Longitudinal evidence further suggests that gambling problems may be associated with subsequent psychiatric difficulties, including anxiety disorders and substance‐related problems.^31^ These findings reinforce the need to integrate gambling assessment within broader mental health evaluations rather than treating gambling behavior as an isolated symptom. The relationship between gambling and cognitive functioning remains unclear. While some studies reported associations between problematic gambling and poorer executive functioning, other findings suggested that recreational gambling may coexist with higher cognitive engagement or social activity.^43^ These differences likely reflect the distinction between recreational and disordered gambling, emphasizing the importance of avoiding the assumption that gambling participation alone represents pathology. Taken together, the evidence partially confirms the hypotheses formulated in the Introduction. Psychosocial vulnerabilities and coping‑related mechanisms consistently emerged across studies, whereas biological contributors were less frequently examined but showed preliminary relevance. This hypothesis‑driven interpretation provides a coherent framework for understanding late‑life gambling and highlights priority areas for future research. Despite the clinical relevance of these findings, intervention research in older adults remains limited. Available evidence suggests that psychological approaches may reduce gambling severity; however, most interventions have not been specifically developed for later life. Future interventions should consider age‐related clinical needs, including social isolation, emotional regulation difficulties, financial consequences, and the role of gambling as a coping behavior. Several limitations of the current evidence base should be considered. First, most included studies used cross‐sectional designs, limiting conclusions regarding causality. Second, the broad age category of 55 years and older may mask important differences between younger‐old and older‐old adults, whose social conditions, health status, and cognitive profiles may differ substantially.^26^, ^45^ Third, the available literature is concentrated in Western countries, limiting generalizability across cultural contexts in which gambling practices and social meanings may differ. In conclusion, this systematic review indicates that Gambling Disorder in older adults is an understudied but clinically meaningful condition. The available evidence suggests that late‐life gambling problems emerge from the interaction between individual vulnerabilities, psychosocial circumstances, and biological factors rather than from aging alone. Clinical approaches should therefore balance recognition of gambling's potential social and recreational functions with careful assessment of situations in which gambling becomes a maladaptive coping strategy. Future research should prioritize longitudinal designs, age‐specific assessment tools, and targeted interventions to improve detection and treatment of Gambling Disorder in later life.

## SCIENTIFIC SIGNIFICANCE

Gambling‐related problems, ranging from at‐risk patterns to diagnosable Gambling Disorder, represent an emerging but still underrecognized concern in older adults. This review highlights key risk factors including social isolation, financial hardship, and emotional dysregulation while emphasizing that these factors should be interpreted within a broader framework of individual vulnerability and age‐related transitions rather than as exclusive characteristics of later life. Although gambling has been more extensively studied in younger populations, evidence regarding older adults remains limited, particularly concerning clinical trajectories, age‐specific assessment, and targeted interventions. Despite preliminary indications that psychological interventions may hold promise, the current evidence base in older adults remains extremely limited and predominantly observational. There is a pressing need for rigorously designed intervention studies specifically targeting this age group. In clinical practice, systematic assessment of gambling‐related problems should be integrated into geriatric assessments, particularly among older adults presenting with loneliness, significant life transitions, financial vulnerability, or psychiatric symptoms. Rather than relying mechanically on standardized tools, clinicians should also consider a functional evaluation of the role that gambling plays in the patient's life, distinguishing, for instance, between a form of socialization and a compulsive escape strategy, since this distinction has direct implications for intervention planning. This approach is particularly important in older adults, in whom gambling may range from a socially embedded leisure activity to a maladaptive coping mechanism associated with significant impairment. Psychological interventions, even when adapted from protocols originally developed for younger adults, appear to hold promise and should be offered wherever available. However, future interventions should incorporate age‐specific targets, including loneliness, emotional regulation, cognitive distortions, and the consequences of retirement or social role changes. From a research perspective, the field urgently requires longitudinal and experimental designs, the development and validation of age‐specific screening instruments, and the systematic inclusion of participants from non‐Western and low‐income contexts, so as to overcome the geographic bias documented in the current literature. Future studies should also adopt age‐stratified approaches, distinguishing “young‐old” from “older‐old” adults, whose risk profiles and clinical presentations may differ substantially. Furthermore, future research should systematically consider biological and medical contributors to gambling vulnerability in later life, including neurological conditions and exposure to medications associated with impulse control disorders, such as dopamine agonists. Finally, at the policy level, gambling regulations should explicitly account for the specific vulnerabilities of older populations while social inclusion programs should be promoted as a structural protective factor. Investment in geriatric mental health infrastructure and in the training of healthcare providers to recognize and address Gambling Disorder in older adults is strongly warranted, particularly given the current underrecognition of this condition in clinical settings.

## CONFLICT OF INTEREST STATEMENT

The authors declare no conflicts of interest.

## Supporting information

Supplementary Table

## Data Availability

Data sharing not applicable to this article as no datasets were generated or analysed during the current study.
