# Supplementary material for: Gambling disorder in late life: A systematic review of behavioral patterns, clinical correlates, and psychotherapeutic interventions
Source: Am J Addict. 2026 Jul 18;35(6):681–701. doi: 10.1111/ajad.70188 (PMC13712548; doi:10.1111/ajad.70188)
Supplement: Supplementary file 1 [file AJAD-35-681-s001.docx]

**Supplementary Table 1.** Search strings and filters applied across electronic databases.

| **Database** | **Search String** |
| --- | --- |
| WOS | TS=("gambling disorder" OR "problem gambling" OR "pathological gambling" OR "compulsive gambling" OR "gambling addiction") AND TS=("older adult*" OR "elder*" OR "late life" OR "aging" OR "geriatric*" OR "senior*" OR "aged") AND TS=("psychotherapy" OR "psychological intervention" OR "cognitive behavioral therapy" OR "CBT" OR "motivational interviewing" OR "psychological treatment") NOT TS=("dementia" OR "Alzheimer*" OR "substance use" OR "alcohol" OR "adolescent*" OR "youth" OR "children" OR "schizophrenia" OR "bipolar" OR "eating disorder") |
| PubMed/MEDLINE | ("behavioral addiction"[MeSH Terms] OR "addiction"[All Fields]) AND ("elderly"[MeSH Terms] OR "older adults"[All Fields] OR “aging population” [ALL Fields] OR “geriatric population” [ALL Fields]) AND ("psychotherapy"[MeSH Terms] OR "psychological interventions"[All Fields]);  ("Gambling"[MeSH Terms] OR "gambling disorder"[All Fields] OR "problem gambling"[All Fields] OR "pathological gambling"[All Fields] OR "compulsive gambling"[All Fields]) AND ("aged"[MeSH Terms] OR "elderly"[All Fields] OR "older adults"[All Fields] OR "late life"[All Fields] OR "aging population"[All Fields] OR "geriatric population"[All Fields]) AND ("psychotherapy"[MeSH Terms] OR "psychological intervention*"[All Fields] OR "cognitive behavioral therapy"[All Fields] OR "CBT"[All Fields] OR "motivational interviewing"[All Fields] OR "psychological treatment*"[All Fields]) |
| Scopus | TITLE-ABS-KEY ("behavioral addiction" OR "addiction") AND ("elderly" OR "older adults" OR “aging population” OR “geriatric population”) AND ("psychotherapy" OR "psychological interventions");   TITLE-ABS-KEY ( ("gambling disorder" OR "problem gambling" OR "pathological gambling" OR "compulsive gambling" OR "gambling behavior*") AND ("elderly" OR "older adults" OR "late life" OR "aging population" OR "geriatric population" OR "seniors") AND ("psychotherapy" OR "psychological intervention*" OR "cognitive behavioral therapy" OR "CBT" OR "motivational interviewing" OR "psychological treatment*") ) |
| PsycNet | ("behavioral addiction" OR "addiction") AND ("older adults" OR "elderly" OR “aging population” OR “geriatric population”) AND ("psychological treatments" OR "psychotherapy");  ("gambling disorder" OR "problem gambling" OR "pathological gambling" OR "compulsive gambling") AND ("older adults" OR "elderly" OR "late life" OR "aging population" OR "geriatric population") AND ("psychological treatment*" OR "psychotherapy" OR "cognitive behavioral therapy" OR "motivational interviewing" OR "psychological intervention*") |
| ClinicalTrials.gov | (“behavioral addiction” AND “elderly” OR “older adults” AND “psychological treatments” OR “psychotherapy”);  ("gambling disorder" OR "problem gambling" OR "pathological gambling")  AND ("elderly" OR "older adults" OR "late life")  AND ("psychotherapy" OR "psychological intervention"  OR "cognitive behavioral therapy") |
| Other Sources:  WHO International Clinical Trials Registry Platform (ICTRP)  Grey Literature (ProQuest Dissertations & Theses Global; SAMHSA; DART-Europe) | ("gambling disorder" OR "pathological gambling" OR "problem gambling" OR "gambling addiction" OR "disordered gambling") AND ("older adult*" OR "elder*" OR "senior*" OR "aged" OR "late-life" OR "55 years" OR "geriatric") AND ("psycholog*" OR "psychotherap*" OR "cognitive behavioural therapy" OR "CBT" OR "intervention*" OR "treatment" OR "risk factor*" OR "correlate*" OR "depression" OR "anxiety" OR "loneliness" OR "cognitive decline") |
